# Therapeutic outcome of early-phase clinical trials in multiple myeloma: a meta-analysis

**DOI:** 10.1038/s41408-021-00441-3

**Published:** 2021-03-01

**Authors:** Niels van Nieuwenhuijzen, Rowan Frunt, Anne M. May, Monique C. Minnema

**Affiliations:** 1 Department of Hematology, University Medical Center Utrecht, Utrecht University, Utrecht, The Netherlands; 2 Center for Translational Immunology, University Medical Center Utrecht, Utrecht University, Utrecht, The Netherlands; 3 Julius Center for Health Sciences and Primary Care, University Medical Center Utrecht, Utrecht University, Utrecht, The Netherlands

**Keywords:** Phase I trials, Phase II trials

## Abstract

Great progress in the treatment of patients with multiple myeloma (MM) has been made due to the development of novel drugs. Patients with relapsed/refractory MM (RRMM) can be enrolled in early-phase clinical trials, but their performance across the last decade is unknown. We conducted a meta-analysis on the overall response rate (ORR) and toxicity. PubMed, Embase, and Cochrane Library were systematically searched for phase I and phase II trials investigating an experimental compound as a single agent or in combination with dexamethasone, published from January 1, 2010 to July 1, 2020. Eighty-eight articles were included, describing 61 phase I trials involving 1835 patients and 37 phase II trials involving 2644 patients. There was a high degree of heterogeneity. Using a random-effects model, the 95% CIs of the estimated ORR were 8–17% for phase I trials and 18–28% for phase II trials. There were significant subgroup differences in ORR between the years of publication in phase I trials and between drug classes in both phase I and phase II trials. The ORR in early-phase clinical trials in RRMM is substantial, especially in phase II trials, but due to high heterogeneity a general assessment of clinical benefit before participation is difficult to offer to patients.

## Introduction

Early-phase clinical trials in oncology are used to determine the safety profile, dosing regimen, and preliminary efficacy of an experimental drug. Phase I trials often represent the first-in-human use and are primarily used to assess toxicities, dosing schedule, and maximum tolerated dose (MTD). Historically, there was no or only very limited therapeutic intent in phase I trials. Phase II trials test the selected dosing regimen in a larger sample to acquire insight into the effectiveness of experimental drugs. In oncology, participants in early-phase clinical trials typically have the advanced-stage disease and have exhausted one or more lines of approved therapeutic regimens. Participants are to expect a higher risk of toxicities, while the activity of the experimental drug is uncertain^1,2^.

In the 1970s and 1980s, overall response rates (ORR) in phase I trials in oncology were estimated to be <5%^3–5^. However, rates of therapeutic success have improved since, with estimated responses of 5–10% in the 1990s and estimates for the past two decades varying from 5 to 20%^6–9^. The American Society of Clinical Oncology, among others, perceives phase I trials as having therapeutic intent and sees participation as a therapeutic option^1,2^. However, it remains subject to debate whether phase I trials genuinely offer therapeutic benefit in relation to their risks^1,2,10–12^.

Multiple myeloma (MM) is a clonal plasma cell malignancy primarily located in the bone marrow and is the second most common hematologic malignancy^13^. There have been many drug approvals for MM in the last 15 years, which have significantly prolonged survival for patients^14^. Despite significant improvements in treatment possibilities and survival, MM remains an incurable malignancy and is characterized by high rates of relapse and therapy resistance^13,15,16^. In the course of disease progression, participation in an early-phase clinical trial can be considered. However, a recent systematic review to inform clinicians and patients on the risks and benefits of these trials is lacking. Therefore, we examined the rates of response and toxicities in early-phase clinical trials in relapsed/refractory MM (RRMM) and assessed differences across years of publication and drug classes.

## Methods

### Search strategy and study selection

This systematic review is reported in accordance with the Preferred Reporting Items for Systematic Reviews and Meta-Analyses (PRISMA) guidelines^17^. Two researchers (NvN and RF) independently searched the publication libraries of PubMed, EMBASE, and the Cochrane Central Register of Controlled Trials from January 1, 2010 to July 1, 2020. Search strings were formulated and verified in accordance with a Utrecht University librarian. The complete search strings are provided in the Supplementary Materials. Searches were limited to publications in the English language. We identified all publications reporting on single-arm phase I and phase II clinical trials with adult RRMM patients that received an experimental compound given as monotherapy or in combination with dexamethasone. Additional relevant articles that were found through other sources were reviewed similarly and could be included when eligibility criteria were met. Studies, where the experimental compound was combined with drugs, other than dexamethasone, were excluded. Studies were also excluded when testing the effect of a drug in a subgroup of RRMM patients, e.g., safety studies in patients with renal failure, or when studies were performed for the mere purpose of approval in one specific country. We further excluded studies related to allogeneic stem cell therapy, bone-directed therapy or supportive care, or studies testing a new route of administration. Studies had to report the activity of the experimental compound on an intention-to-treat population of at least ten RRMM patients.

### Data extraction

Results were extracted from the included studies and tabulated by two researchers (NvN and RF) independently. Potential conflicts were resolved by discussion with a third researcher (MCM). In case of missing data, corresponding authors of the studies were contacted. When we identified multiple publications on the same study population, results from the most recent and/or most complete original publication were used for data extraction. When not all data could be extracted from the original article, a trial registration database (ClinicalTrials.gov) was used to find additional relevant publications in order to obtain missing data. For studies that reported the results of a combined phase I and phase II trial, we manually extracted data of patients belonging to the respective trial phase.

The primary outcome was the ORR, defined as a best-reported partial response (PR) or better. Secondary outcomes were toxicities, measured as dose-limiting toxicities (DLTs) and drug discontinuations, and the clinical benefit rate (CBR): a best-reported response of minimal response (MR) or better^18^. We included CBR since also a minor response can be a signal of single-drug activity and may have additional value to RRMM patients^19^. From each included study, we extracted the following characteristics: trial stage, Clinical Trial Registration Number, name, type, and a class of experimental drug, intention-to-treat population, median lines of prior therapy, response rates: ORR (≥PR) and CBR (≥MR), toxicity profile: number of DLTs or number of patients discontinuing the trial drug due to adverse events. Wherever possible, we extracted the reported response rates according to the International Myeloma Working Group definitions^18^.

### Statistical analysis

We used statistical software R, version 4.0.0, for all data analyses^20^. The package “meta” (4.12-0) was used for meta-analyses^21^. We applied logit transformations to turn the proportions into normal distributions suitable to pool effects. The weight of each included study in the meta-analysis was determined with the generic inverse variance method. This means that larger studies, which generally have smaller variances than small studies, will be given a relatively greater weight within the meta-analysis. Effect sizes from each study were then pooled with a random-effects model. Preplanned subgroup analyses were devised for years of publication and drug class. To evaluate the degree of statistical heterogeneity, we used the Cochran Q statistic and inconsistency index (*I*^2^). Heterogeneity was classified as low (*I*^2^ = 25%), moderate (*I*^2^ = 50%), and high (*I*^2^ = 75%)^22^. All effect sizes are reported with corresponding 95% confidence intervals (CI) and prediction intervals (PI). Reported *P* values are two-sided, with a *P* < 0.05 considered significant. We used the test for subgroup differences to investigate how the estimates vary across subgroups of years of publication and drug classes.

## Results

### Selection of studies

**Fig. 1:**
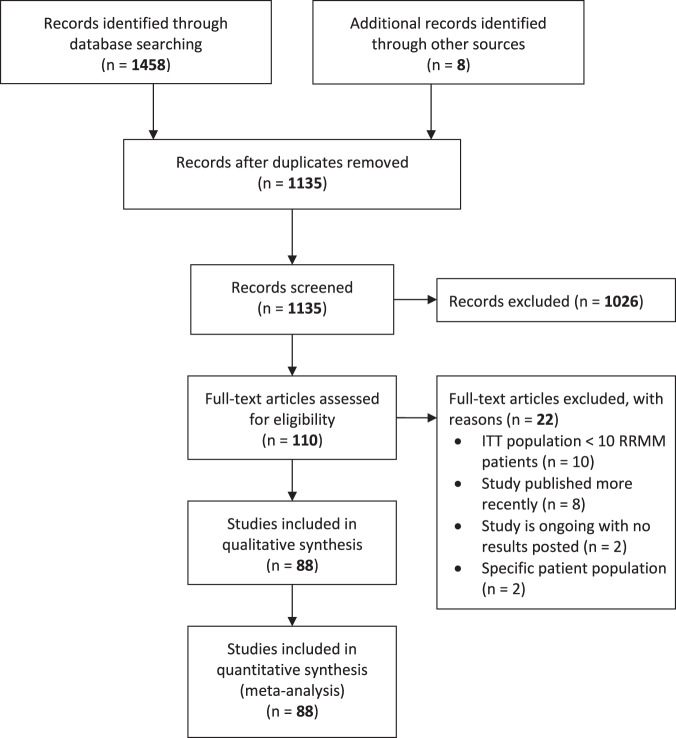
PRISMA flow diagram of the study selection process. ITT intention-to-treat, RRMM relapsed/refractory multiple myeloma.

Our literature search identified 1127 original publications for review after the removal of duplicates. A further eight articles were found through other sources and added to the selection, making the total 1135 publications. After screening on title and abstract, 110 potentially relevant articles were selected for full-article review on eligibility. Of these, 88 studies were included for data extraction and quantitative analysis (Supplementary eTable 1)^23–112^. The selection process is visualized in Fig. 1.

### Study and patient characteristics

The 88 studies that were included for analysis together report the results of 98 clinical trials, of which 61 phase I trials and 37 phase II trials. Several publications reported the results of both a phase I and phase II trial. In total, 4479 patients were included, of which 1835 in phase I trials and 2644 in phase II trials (Supplementary eTable 2).

We divided all included studies into four subgroups based on their year of publication: 2010–2012 (*n* = 16), 2013–2015 (*n* = 22), 2016–2018 (*n* = 35), and 2019–2020 (*n* = 26) (Table 1). In addition, we subdivided the studies into nine different subgroups based on the drug class of the experimental compound: immunomodulatory imide drugs (IMiDs) (*n* = 4), proteasome inhibitors (*n* = 20), monoclonal antibodies (*n* = 18), cell therapy (*n* = 5), antibody–drug conjugates (*n* = 7), kinase inhibitors (*n* = 10), immune checkpoint inhibitors (*n* = 2), heat-shock protein 90 inhibitors (*n* = 5), and others (*n* = 27). Thirty-five (58%) of phase I trials and 29 (78%) of phase II trials reported on the median lines of prior therapies received by patients before participation in the trial. Median lines of prior therapies ranged from two to more than seven, while patients in phase I trials seem to have been more heavily pretreated than patients in phase II trials (Table 1). In 39 (40%) trials, dexamethasone was given in addition to the novel compound from the start of the trial. Most trials were designed specifically for RRMM; five trials were set up to test the experimental drug in other malignancies as well.

**Table 1:** Included studies per subgroup.

	Phase I, *N* = 61 No. (%)	Phase II, *N* = 37 No. (%)	Total, *N* = 98 No. (%)
*Years*			
2010–2012	9 (15)	7 (19)	16 (16)
2013–2015	14 (23)	8 (22)	22 (22)
2016–2018	22 (36)	13 (35)	35 (36)
2019–2020	16 (26)	9 (24)	25 (26)
*Drug*			
IMiD	1 (2)	3 (8)	4 (4)
PI	9 (15)	11 (30)	20 (20)
mAb	12 (20)	6 (16)	18 (18)
Cell therapy	6 (10)	–	6 (6)
ADC	6 (10)	1 (3)	7 (7)
Kinase inhibitor	5 (8)	5 (14)	10 (10)
ICI	2 (3)	–	2 (2)
Hsp90i	5 (8)	–	5 (5)
Other	15 (25)	11 (30)	26 (27)
*Median lines of prior therapy*			
1–3	7 (11)	9 (24)	16 (16)
4-6	22 (36)	18 (49)	40 (41))
≥7	7 (11)	2 (5)	9 (9)
Unknown	25 (41)	8 (22)	33 (34)

*IMiD* immunomodulatory imide drugs, *PI* proteasome inhibitors, *mAb* monoclonal antibodies, *ADC* antibody–drug conjugate, *ICI* immune checkpoint inhibitor, *Hsp90i* heat-shock protein 90-inhibitor.

### Response rate

**Fig. 2:**
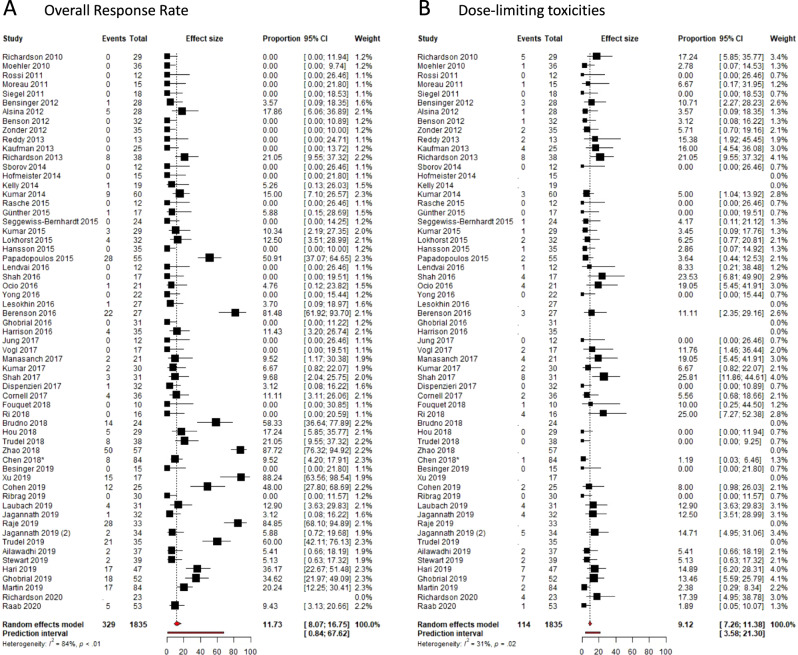
Forest plots of overall response rates and dose-limiting toxicities in phase I trials. **A** Overall response rates per included phase I trial. **B** Drug-limiting toxicities per included phase I trial. Forest plots are arranged by the population size of trials within each year of publication. Squares represent estimated proportions, with the size of the squares representing the weight of each trial according to the inverse variance method. Horizontal lines through the squares indicate 95% CIs. The points of the diamond indicate the 95% CI of the pooled mean. The horizontal line at the bottom indicates the prediction interval. Blank lines indicate missing information from the trial paper. *The study population in Chen et al.^84^ included two patients with Waldenström’s Macroglublinemia.

**Fig. 3:**
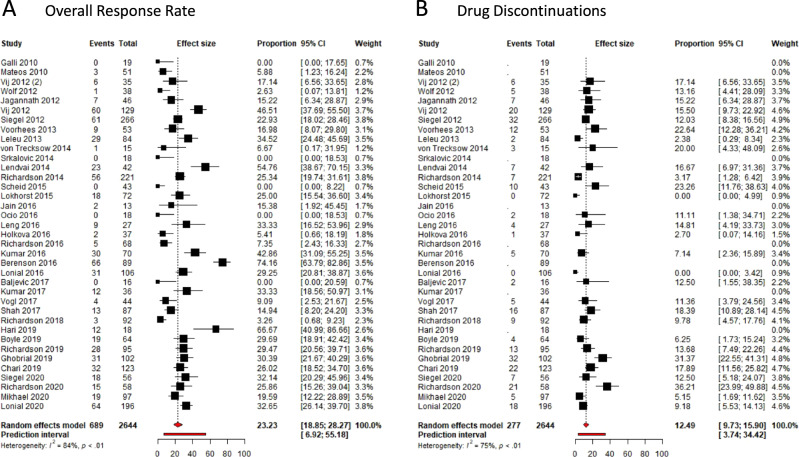
Meta-analysis of overall response rates and dose-limiting toxicities in phase II trials. **A** Overall response rates per included phase II trial. **B** Drug discontinuations - per included phase II trial. Forest plots are arranged by the population size of trials within each year of publication. Squares represent estimated proportions, with the size of the squares representing the weight of each trial according to the inverse variance method. Horizontal lines through the squares indicate 95% CIs. The points of the diamond indicate the 95% CI of the pooled mean. The horizontal line at the bottom indicates the prediction interval. Blank lines indicate missing information from the trial paper.

The ORR to the experimental drug was reported in all but one of the included trials. The degree of heterogeneity between the studies for ORR was high in both phase I (*I*^2^ = 84%, *P* < 0.001) and phase II studies (*I*^2^ = 84%, *P* < 0.001). The 95% CI of the estimated overall response was 8.1–16.8% for all included phase I trials and 18.9–28.3% for all included phase II trials. The prediction interval of the estimated overall responses was 0.8–67.6% for phase I trials and 6.9–55.2% for phase II trials (Figs. 2 and 3).

**Fig. 4:**
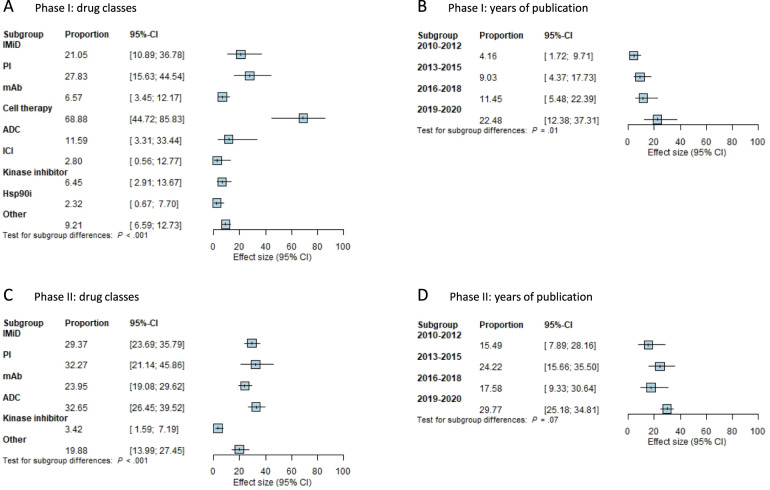
Estimates of overall response rates for subgroups. **A** Overall response rates in phase I trials by drug class. **B** Overall response rates in phase I trials by years of publication. **C** Overall response rates in phase II trials by drug class. **D** Overall response rates in phase II trials by years of publication. Squares represent estimate values. Horizontal lines through the squares indicate 95% CIs. IMiD immunomodulatory imide drugs, PI proteasome inhibitors, mAb monoclonal antibodies, ADC antibody–drug conjugate, ICI immune checkpoint inhibitor, Hsp90i heat-shock protein 90-inhibitor.

We examined possible associations between the estimated response and subgroups of trials based on the year of publication. Subgroup analysis revealed a significant difference between years of publication in phase I trials (*P* = 0.01), but not in phase II trials (*P* = 0.07) (Fig. 4). In phase I trials, the 95% CI of the response estimate of trials published from January 1, 2010 to December 31, 2012 was 1.7–9.7%, while for trials published from January 1, 2019 to July 1, 2020 the 95% CI was 12.4–37.3%. Furthermore, ORR estimates varied according to the drug class of the investigational compound. The test for subgroup differences demonstrated a significant difference between drug classes for both phase I (*P* < 0.0001) and phase II (*P* < 0.0001) trials (Fig. 4). In phase I trials, the 95% CI of the response estimates were low for drug classes Hsp90i (0.7–7.7%) and kinase inhibitors (2.9–13.7%), and high for drug classes PI (15.6–44.5%) and cell therapy (44.7–85.8%). Since most of the CAR T-cell trials were published in 2019, these trials seem to be responsible for the association between ORR and the year of publications (Supplementary eTable 3)^113,114^.

### Toxicities

The rate of DLTs in phase I trials and rate of drug discontinuations in phase II trials were collected as surrogate markers of important drug-related toxicities. We were able to gather the number of DLTs for 51 of 60 phase I trials and the number of drug discontinuations in 29 out of 37 clinical trials. For phase I trials, there was an intermediate degree of heterogeneity (*I*^2^ = 31.5%, *P* = 0.02). The estimated proportion of DLTs was 9.1% (95% CI 7.3–11.4%). The prediction interval was 3.6–21.3%. (Fig. 2). For phase II trials, heterogeneity was scored as borderline intermediate (*I*^2^ = 74.6%, *P* < 0.001). The estimated overall number of drug discontinuations was 12.49% (95% CI 9.7–15.9%), with the prediction interval ranging from 3.7 to 34.4% (Fig. 3).

For the estimate of DLTs in phase I trials, we did not find any subgroup differences for both years of publication (*P* = 0.77) and drug class (*P* = 0.10). For drug discontinuations in phase II trials, there were significant subgroup differences for drug class (*P* = 0.03) but not for years of publication (*P* = 0.55).

### Clinical benefit rate

The CBR was reported in 48 of 60 phase I trials and 30 of 37 phase II trials. Again, heterogeneity between individual studies was high for both phase I (*I*^2^ = 79%, *P* < 0.001) and phase II trials (*I*^2^ = 86%, *P* < 0.01). The 95% CI of the estimated overall CBR was 13.7–24.1% for phase I trials, including 1456 patients, and 28.7–40.4% for phase II trials, involving 2397 patients. The prediction interval of the estimated overall CBR was 3.0–62.4% for phase I trials and 12.4–65.8% for phase II trials (Supplementary eFig. 1).

Similar to the ORR, we analyzed subgroup differences across different years of publication and different drug classes. The test for subgroup differences was significant for years of publication for phase I (*P* = 0.01) but not phase II (*P* = 0.2) trials. In addition, there was a significant difference between drug classes for both phase I (*P* < 0.001) and phase II (*P* < 0.011) studies.

## Discussion

Patients with RRMM will inevitably exhaust multiple lines of approved treatment regimens since it currently remains an incurable disease. In specific trial centers, fit patients are offered enrollment in early-phase clinical trials, but contemporary data on the performance and toxicities of these types of trials is lacking for MM^1,2,10^. In this systematic review and meta-analysis of early-phase clinical trials, we assessed the potential benefit and risks to patients across the past decade.

We searched for trials that investigate single-drug activity in RRMM because these types of drugs are most likely to be beneficial to patients in follow-up randomized phase III studies. Since dexamethasone is so frequently used, also to prevent side effects of the experimental drug, we did allow for its use in the current analysis. In total, we found 88 original studies, which is a considerable number of studies for a meta-analysis of early-phase clinical trials in one specific disease^6–8^.

The heterogeneity between the individual studies was high, with *I*^2^ consistently above 75%. This was to be expected with the inclusion of trials testing therapy with widely varying modes of action. Therefore, we considered it inappropriate to provide a single estimate of the response rates as a mean of all early-phase trials. Instead, we provided the 95% CI of the estimated mean and the prediction intervals. The 95% CI presents a summary of the treatment effect, while the prediction interval shows the range of effects that can be expected in future similar studies. The prediction interval implies that the ORR of a future phase I trial may range from 0.8% all the way up to 67.6%, based on results from the past decade (Fig. 2). The 95% CI of the estimated response rate of phase I trials was 8.1–16.8%, which is comparable to recent analyses on therapeutic effectivity of phase I trials in oncology for non-hematological malignancies^8,9^.

Our results show that response rates are dependent on the drug class of the experimental compound (Figs. 2 and 4). Response rates of approved and currently widely used drugs such as PI or IMiDs are better than drugs such as Hsp90-inhibitors or kinase inhibitors. The latter has failed to demonstrate activity as a single agent and have not progressed to phase III trials. Notably, anti-B Cell Maturation Antigen (BCMA) CAR T cells have shown remarkable activity in relapsed MM patients with a 95% CI of 44.5–85.8%^113,114^. However, performing the meta-analysis without these trials only reduced heterogeneity by several percentage points (Supplementary eTable 3). Currently, results have only been published for phase I studies of anti-BCMA CAR T cells, but phase II results are expected with great anticipation.

Patients with active RRMM can also benefit from a treatment that merely prevents further disease progression and thereby avoids the development of myeloma-related symptoms. Therefore, we analyzed the CBR of the included trials. A MR indicates that the drug used as monotherapy has some activity in the disease under investigation and may be suitable for combination strategies in the future. Effect estimates of the CBR (phase I: 13.7–24.1%; phase II: 28.7–40.4%) were higher than for the ORR (phase I: 8.1–16.8%; phase II: 18.9–28.3%), while the range of the prediction interval of the CBR was comparable to the prediction interval of the ORR (Fig. 2 and Supplementary eFig. 1).

As a surrogate marker for important drug-related toxicities, we chose the rate of DLTs in phase I trials and drug discontinuations in phase II trials. DLTs are standardly described in phase I trial protocols and are used to establish the MTD of the experimental drug. There was a consistent rate of DLTs in phase I studies of 9.1% and a rate of drug discontinuations of 12.5% in phase II studies (Fig. 2). With the advent of targeted therapies and immune therapies, the linear relationship between dose and efficacy is less apparent and the same holds true for the relationship between dose and toxicities.

The trials that are pooled are heterogeneous due to the different interventions used. A major limitation of our study is the considerable heterogeneity that exists between individual trials as a result of the prior drug exposure of patients. This is reflected by differences in the median line of therapies received before participating in a trial, but the response to the experimental drug also depends on the type of drugs a patient has received at initial diagnosis and subsequent relapses. Therapy regimens have not only changed considerably over the past decade but are also dependent on what region of the world the patient is treated in. Therefore, trial results are dependent on how, when, and where a patient was treated previously. As such the heterogeneity in patient populations between trials poses limitations on the generalizability of our findings. Another shortcoming of early-phase clinical trials is that the primary outcome is the best response, which is a surrogate marker for improved overall survival (OS). Although ORR and OS are associated, a higher ORR might not necessarily implicate an improvement in OS^115^. Progression-free survival and OS are better measurements of clinical benefit but are not generally reported for early-phase clinical trials.

There are several factors that may contribute to a further increase in the efficacy of early-phase clinical trials in the near future. Part of the heterogeneity between studies was due to the high ORR of studies testing anti-BCMA CAR T cells^114^. These and other promising novel forms of immune therapy may substantially increase response rates without increasing toxicity. Another step that may improve therapeutic success is an intrapatient dose escalation, which helps patients to receive safe and higher doses of a potentially active novel drug. In addition, biomarker selection can improve the selection process of patients likely to benefit from a novel drug^116^.

## Conclusions

The increased understanding of cancer pathobiology—and MM especially—has resulted in many novel drugs that specifically target malignant cells. Immunotherapies have been developed for MM patients and have opened a completely new set of treatment possibilities. Consequently, response rates in early-phase clinical trials have improved over the past decades. In this meta-analysis, we characterized the overall effect of participating in early-phase clinical trials for RRMM patients over the past decade. We found that the ORR of early-phase clinical trials is highly variable, but seems to have improved over the past decade without a concurrent rise in toxicities. These improvements are mainly due to novel classes of therapy, such as PIs, IMiDs, and CAR T cells. Our findings can be used to facilitate debate on the risks and benefits of participation in early-phase clinical trials in oncology in general and may be used by clinicians to guide assessment and communication on enrollment of RRMM patients in early-phase clinical trials.

## Supplementary information

supplementary content
