# Supplementary material for: Therapeutic outcome of early-phase clinical trials in multiple myeloma: a meta-analysis
Source: Blood Cancer J. 2021 Mar 1;11(3):44. doi: 10.1038/s41408-021-00441-3 (PMC7921415; doi:10.1038/s41408-021-00441-3)
Supplement: Supplementary file 1 — supplementary content [file 41408_2021_441_MOESM1_ESM.docx]

**Supplementary content**

Supplementary Table 1. Included Studies and Characteristics

Supplementary Table 2. Included Patients per Subgroup

Supplementary Figure 1. Forest Plots of Clinical Benefit Rate in Phase I and Phase II trials

Supplementary Table 3. Results with sequential omittance of single subgroup

Search strings

**Supplementary Table 1.** **Included Studies and Characteristics**

| **PMID** | **First author** | **Year** | **Clinical Trial registration number** | **Drug** | **Trial phase** | **N (ITT)** | **Including dexamethasone from start** | **Other malignancies** |
| --- | --- | --- | --- | --- | --- | --- | --- | --- |
| 31588567 | Siegel | 2020 | NCT01946477 | Pomalidomide | II | 56 | + | - |
| 31859245 | Lonial | 2020 | NCT03525678 | Belantamab mafodotin | II | 196 | - | - |
| 32171061 | Raab | 2020 | NCT01421186 | MOR202 | I/II | 53 | + | - |
| 32213344 | Richardson | 2020 | NCT01897714 | Melflufen | I/II | 81 | + | - |
| 32409691 | Mikhael | 2020 | NCT01084252 | Isatuximab | II | 97 | - | - |
| 31218679 | Boyle | 2019 | NCT02626481 | Daratumumab | II | 64 | + | - |
| 31142508 | Ghobrial | 2019 | NCT01416428 | Oprozomib | I/II | 154 | + | - |
| 31229804 | Hari | 2019 | NCT01832727 | Oprozomib | I/II | 65 | + | - |
| 30896447 | Cohen | 2019 | NCT02546167 | CAR T-BCMA | I | 25 | - | - |
| 30930134 | Jagannath | 2019 | NCT00723359/NCT01001442 | Indatuximab ravtansine | I/II | 66 | - | - |
| 30988175 | Xu | 2019 | NCT03090659 | CAR T-BCMA (LCAR-B38M) | I | 17 | - | - |
| 31042825 | Raje | 2019 | NCT02658929 | CAR T-BCMA  bb2121 | I | 33 | - | - |
| 30894515 | Trudel | 2019 | NCT02064387 | GSK2857916 | I | 35 | + | - |
| 30926770 | Martin | 2019 |  | Isatuximab | I | 84 | - | - |
| 30718503 | Stewart | 2019 | NCT01432353 | DFRF4539A | I | 39 | - | - |
| 30279233 | Laubach | 2019 | NCT01522872 | Evofosfamide | I/II | 31 | + | - |
| 30340993 | Ailawadhi | 2019 | NCT00346255 | lorvotuzumab mertansine | I | 37 | - | - |
|  | Besinger | 2019 | NCT03340883 | BION-1301 | I | 15 | - | - |
| 31433920 | Chari | 2019 | NCT02336815 | Selinexor | II | 123 | + | - |
| 30937889 | Ribrag | 2019 | NCT01953692 | Pembrolizumab | I | 30 | - | - |
|  | Richardson | 2019 | NCT02963493 | Melflufen | II | 95 | + | - |
| 29812997 | Brudno | 2018 | NCT02215967. | CAR T-BCMA | I | 24 | - | - |
| 30572922 | Zhao | 2018 | NCT03090659 | CAR T-BCMA (LCAR-B38M) | I | 57 | - | - |
| 30442502 | Trudel | 2018 | NCT02064387 | GSK2857916 | I | 38 | + | - |
| 28930776 | Hou | 2018 | ChiCTR-TNRC-12001896 | Circularly permuted TRAIL | I | 29 | - | - |
| 29844860 | Fouquet | 2018 |  | F50067 | I | 10 | - | - |
| 29435979 | Richardson | 2018 | NCT01478581 | Ibrutinib | II | 92 | + | - |
| 29203585 | Chen | 2018 | NCT01607892 | Selinexor | I | 84: 81 MM en 3 WM | + | +  WM |
| 29225263 | Ri | 2018 | JapicCTI-111652 | NK012 | I | 16 | + | - |
| 28817190 | Shah | 2017 | NCT00821249 | Filanesib | I/II | 118 | + | - |
| 29018077 | Kumar | 2017 | NCT01794520 | Venetoclax | I/II | 66 | + | - |
| 28140719 | Vogl | 2017 | NCT01023880 | Delanzomib | I/II | 61 | - | - |
| 28337527 | Baljevic | 2017 | NCT01447914 | Tivantinib | II | 16 | - | - |
| 28088784 | Jung | 2017 | NCT02248402 | Vax-DC | I | 12 | - | - |
| 28439108 | Dispenzieri | 2017 | NCT00450814 | MV-NIS | I | 32 | - | - |
|  | Manasanch | 2017 | NCT02426723 | CWP232291 | I | 21 | - | - |
|  | Cornell | 2017 | NCT02649790 | Eltanexor | I/II | 36 | + | - |
| 27702799 | Kumar | 2016 | NCT01415882 | Ixazomib | II | 70 | + | - |
| 27117181 | Harrison | 2016 | NCT00629473 | Marizomib | I | 35 | + | - |
| 27269947 | Lesokhin | 2016 | NCT01592370 | Nivolumab | I | 27 | - | +  NHL, HL, CLL |
| 27207788 | Berenson | 2016 | NCT01677858 | Carfilzomib | I/II | 116 | + | - |
| 27009059 | Richardson | 2016 | NCT00461045 | Marizomib | II | 68 | + | - |
| 26446942 | Holkova | 2016 | NCT01085214 | Selumetinib | II | 37 | - | - |
| 26695442 | Yong | 2016 | NCT00457782 | KW-2478 | I | 22 | - | - |
| 26561559 | Shah | 2016 | NCT00722488 | Pevonedistat | I | 17 | - | - |
| 27581088 | Jain | 2016 | NCT01646762 | Nab‐paclitaxel | II | 13 | - | - |
| 27608772 | Leng | 2016 | ChiCTR-ONC-12002065 | Circularly permuted TRAIL | II | 27 | - | - |
| 27020089 | Lendvai | 2016 | NCT01866293/NCT01582295 | Cabozantinib | I | 12 | - | - |
| 26800393 | Ghobrial | 2016 | NCT01118689 | TAK-228 | I | 31 | - | +  NHL, WM |
| 26033438 | Ocio | 2016 |  | PM00104 | I/II | 39 | + | - |
| 26778538 | Lonial | 2016 | NCT01985126 | Daratumumab | II | 106 | - | - |
| 25402977 | Scheid | 2015 |  | Dovitinib | II | 43 | + | - |
| 25712687 | Hansson | 2015 | NCT01025206 | BI-505 | I | 35 | - | - |
| 25395429 | Kumar | 2015 | NCT01096342 | Dinaciclib | I/II | 29 | + | - |
| 25637055 | Rasche | 2015 | NCT01727778 | PAT-SM6 | I | 12 | - | - |
| 25682600 | Günther | 2015 | 2006-002675-41 (EudraCT) | Everolimus | I | 17 | - | - |
| 26308596 | Lokhorst | 2015 | NCT00574288 | Daratumumab | I/II | 104 | - | - |
| 25809731 | Seggewiss-Bernhardt | 2015 | NCT00708292 | NVP-AUY922 | I/II | 24 | - | - |
| 25225420 | Papadopoulos | 2015 | NCT00531284 | Carfilzomib | I | 55 | + | - |
| 25294913 | Sborov | 2014 | NCT01533194 | Reolysin | I | 12 | - | - |
| 24913924 | Srkalovic | 2014 | NCT00253578 | Sorafenib | II | 18 | - | - |
| 24904120 | Kumar | 2014 | NCT00963820 | Ixazomib | I | 60 | - | - |
| 24963043 | Lendvai | 2014 | NCT01351623 | Carfilzomib | II | 42 | + | - |
| 24421329 | Richardson | 2014 | NCT00833833 | Pomalidomide | II | 221 | + | - |
| 24241210 | Hofmeister | 2014 | NCT00112723 | Flavopiridol | I | 15 | - | - |
| 24352795 | Kelly | 2014 | NCT00697346 | MLN8237 | I | 19 | - | +  NHL, CLL |
| 23713484 | Von Tresckow | 2014 | NCT00368121 | Cetuximab | II | 15 | + | - |
| 24112026 | Kaufman | 2013 | NCT00421525 | Milatuzumab | I | 25 | - | - |
| 23763921 | Reddy | 2013 | NCT00595686 | PF-04929113 | I | 13 | - | +  DLBCL, FL, CLL, AML, HL |
| 23432640 | Voorhees | 2013 |  | Siltuximab | II | 53 | + | - |
| 23243282 | Richardson | 2013 | NCT00833833 | Pomalidomide | I | 38 | + | - |
| 23319574 | Leleu | 2013 | NCT01053949 | Pomalidomide | II | 84 | + | - |
| 23033266 | Benson | 2012 | NCT00552396 | Anti-KIR | I | 32 | - | - |
| 22833546 | Siegel | 2012 | NCT00511238 | Carfilzomib | II | 266 | + | - |
| 23040437 | Jagannath | 2012 | NCT00511238 | Carfilzomib | II | 46 | + | - |
| 22861192 | Bensinger | 2012 | NCT00231166 | HCD122 | I | 28 | + | - |
| 22761464 | Alsina | 2012 | NCT00150462 | Carfilzomib | I | 28 | + | - |
| 22845873 | Vij | 2012 | NCT00530816 | Carfilzomib | II | 35 | + | - |
| 22184404 | Zonder | 2012 |  | Elotuzumab | I | 35 | - | - |
| 22555973 | Vij | 2012 | NCT00530816 | Carfilzomib | II | 129 | + | - |
| 22288662 | Wolf | 2012 |  | Panobinostat | II | 38 | - | - |
| 21851215 | Siegel | 2011 |  | IPI-504 | I | 18 | - | - |
| 21321571 | Moreau | 2011 |  | AVE1642 | I | 15 | - | - |
| 21454215 | Rossi | 2011 |  | Atacicept | I | 12 | - | - |
| 19633847 | Galli | 2010 |  | ITF2357 | II | 19 | + | - |
| 20530693 | Mateos | 2010 | NCT00229203 | Plitidepsin | II | 51 | + | - |
| 20571355 | Moehler | 2010 |  | Imexon | I | 36 | - | - |
| 20618337 | Richardson | 2010 |  | Tanespimycin | I | 29 | - | - |

Supplementary Table 1.

Title: Included Studies and Characteristics

Legend: ITT, intention-to-treat; WM, Waldenström’s macroglobulinemia; NHL, non-Hodgkin lymphoma; CLL, chronic lymphocytic leukemia; DLBCL, diffuse large B-cell lymphoma; FL, follicular lymphoma; AML, acute myeloid leukemia; HL, Hodgkin lymphoma

**Supplementary Table 2. Included Patients per Subgroup**

|  |  | **Phase I**  **N = 1835**  **No. (%)** | **Phase II**  **N = 2644**  **No. (%)** | **Total**  **N = 4479**  **No. (%)** |
| --- | --- | --- | --- | --- |
| **Years** | **2010-2012** | 233 (13) | 584 (22) | 817 (18) |
|  | **2013-2015** | 386 (21) | 548 (21) | 934 (21) |
|  | **2016-2018** | 629 (34) | 703 (27) | 1332 (30) |
|  | **2019-2020** | 587 (32) | 809 (31) | 1396 (31) |
| **Drug** | **IMiD** | 38 (2) | 361 (14) | 399 (9) |
|  | **PI** | 333 (18) | 909 (34) | 1242 (28) |
|  | **mAb** | 376 (21) | 407 (15) | 783 (18) |
|  | **Cell therapy** | 168 (9) | - | 156 (3) |
|  | **ADC** | 215 (12) | 196 (7) | 411 (9) |
|  | **Kinase inhibitor** | 111 (6) | 206 (8) | 317 (7) |
|  | **ICI** | 57 (3) | - | 57 (1) |
|  | **Hsp90i** | 106 (6) | - | 106 (2) |
|  | **Other** | 431 (24) | 565 (21) | 996 (22) |

Supplementary Table 2

Title: Included Patients per Subgroup

Legend: IMiD, Immunomodulatory imide drugs; PI, proteasome inhibitors; mAb, monoclonal antibodies; ADC, antibody-drug conjugate; ICI, immune checkpoint inhibitor; Hsp90i, Heat-shock protein 90-inhibitor

**
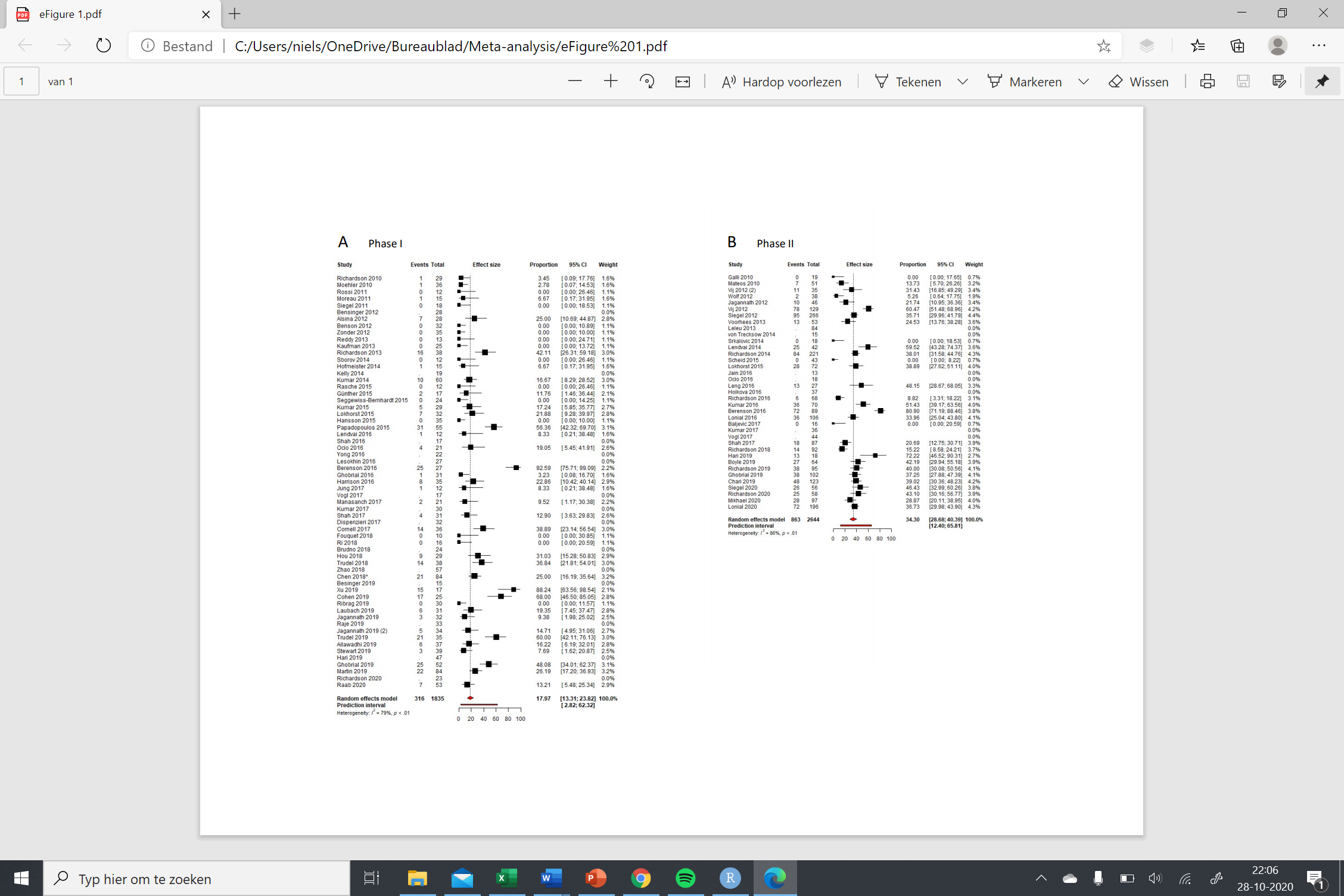
****Supplementary Figure 1. Forest Plots of Clinical Benefit Rate in Phase I and Phase II trials**

Supplementary Figure 1

Title: Forest Plots of Clinical Benefit Rate in Phase I and Phase II trials

Legend: A, Clinical benefit rates per included phase I trial. B, Clinical benefit rate per included phase II trial.

Forest plots are arranged by the population size of trials within each year of publication. Squares represent estimated proportions, with the size of the squares representing the weight of each trial according to the inverse variance method. Horizontal lines through the squares indicate 95% CIs. The points of the diamond indicate the 95% CI of the pooled mean. Horizontal line at the bottom indicates the prediction interval. Blank lines indicate missing information from the trial manuscript.

**Supplementary Table 3. Results with sequential omittance of single subgroup**

**A. Phase I without each respective drug class**

|  | CARs | ADC | mAb | Kinase | ICI | PI | IMiD | Hsp90i | other |
| --- | --- | --- | --- | --- | --- | --- | --- | --- | --- |
| k | 54 | 54 | 48 | 55 | 58 | 51 | 59 | 55 | 45 |
| REM | 9.15 (6.48 -12.76) | 11.44 (7.68 -16.71) | 13.48 (8.92 -19.87) | 12.27 (8.35 - 17.67) | 12.00 (8.26 - 17.13) | 9.19 (5.88 -14.09) | 11.25 (7.67 - 16.22) | 12.67 (8.70 - 18.09) | 13.50 (8.77 - 20.21) |
| I^2^ (%) | 75.5 | 83.1 | 85 | 84.2 | 83.6 | 82.3 | 83.7 | 84.1 | 85.4 |
| Subgroup an. (years) | 0,16 | 0,0015 | 0,088 | 0,015 | 0,0065 | 0,0061 | 0,010 | 0,040 | 0,028 |
| 10-12 (I^2^ %) | 28.9 | 28.9 | 44.4 | 28.9 | 28.9 | 0.0 | 28.9 | 37.9 | 37.9 |
| 13-15 | 75.3 | 75.3 | 77.9 | 79 | 75.3 | 16.2 | 77.2 | 76.7 | 76.4 |
| 16-18 | 71.8 | 86.3 | 86.2 | 86 | 86 | 84.1 | 85.7 | 86.1 | 89.6 |
| 19-20 | 82.7 | 87.5 | 88.2 | 87.8 | 88.1 | 89.4 | 87.8 | 87.8 | 88.2 |

**B. Phase I without each respective block of years of publication**

|  | 10-12 | 13-15 | 16-18 | 19-20 |
| --- | --- | --- | --- | --- |
| k | 51 | 46 | 38 | 45 |
| REM | 13.4919 (9.1736 - 19.4085) | 12.2790 [ 7.9014; 18.5923] | 11.9004 [ 7.6672; 18.0149] | 8.4756 [ 5.3065; 13.2720] |
| I^2^ (%) | 84.6 | 85.1 | 82.2 | 80.4 |
| Subgroup an. (years) | 0,10 | 0,0055 | 0,0045 | 0,21 |
| 10-12 (I^2^ %) |  | 28.9 |  |  |
| 13-15 | 75.3 |  |  |  |
| 16-18 | 85.7 |  |  |  |
| 19-20 | 87.8 |  |  |  |

Legend: IMiD, Immunomodulatory imide drugs; PI, proteasome inhibitors; mAb, monoclonal antibodies; ADC, antibody-drug conjugate; ICI, immune checkpoint inhibitor; Hsp90i, Heat-shock protein 90-inhibitor

**Search strings**

Pubmed

("Multiple Myeloma"[Mesh] OR Multiple Myeloma[Title/Abstract]) AND (relapse[Title/Abstract] OR relapsed[Title/Abstract]) AND (Clinical Trial, Phase I[ptyp] OR Clinical Trial, Phase II[ptyp]) AND ("2010/01/01"[PDat] : "2020/12/31"[PDat])

EMBASE

('multiple myeloma'/exp OR 'multiple myeloma*':ti,ab,kw) AND (relapse:ti,ab,kw OR relapsed:ti,ab,kw) AND ('phase 1 clinical trial'/de OR 'phase 1 clinical trial (topic)'/de OR 'phase 2 clinical trial'/de OR 'phase 2 clinical trial (topic)'/de) AND (2010:py OR 2011:py OR 2012:py OR 2013:py OR 2014:py OR 2015:py OR 2016:py OR 2017:py OR 2018:py OR 2019:py OR 2020:py) AND [embase]/lim AND 'Article'/it

CENTRAL

*ID Search*

#1 ("multiple myeloma"):ti,ab,kw (Word variations have been searched)

#2 ("relapse"):ti,ab,kw (Word variations have been searched)

#3 ("relapsed"):ti,ab,kw (Word variations have been searched)

#4 #2 OR #3

#5 clinical trial, phase I in Trials

#6 clinical trial, phase II in Trials

#7 clinical trial, phase 1 in Trials

#8 clinical trial, phase 2 in Trials

#9 #5 OR #6 OR #7 OR #8

#10 #1 AND #4 AND #9 with Publication Year from 2010 to 2020, in Trials
